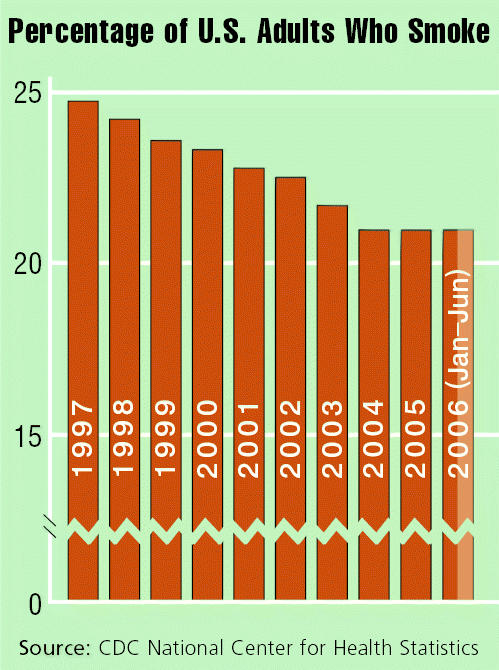# The Beat

**Published:** 2007-02

**Authors:** Erin E. Dooley

## Dirty City Roundup

In October 2006, the Blacksmith Institute released a report identifying the world’s 10 most polluted cities based on factors such as toxicity of the pollution involved and clear evidence of health impacts. In Dzherzhinsk, Russia, which was a chemical weapons manufacturing site during the Cold War, people have a life expectancy about half that of people in the world’s richest nations. Linfen, China, another of the top 10, lies in the heart of that country’s coal-producing Shanxi Province; its residents suffer from bronchitis, pneumonia, and lung cancer thought to be the result of the area’s poor air quality. The other eight cities are Norilsk and Rudnaya Pristan, Russia; Haina, Dominican Republic; Ranipet, India; Mailuu-Suu, Kyrgyzstan; La Oroya, Peru; Chernobyl, Ukraine; and Kabwe, Zambia.

**Figure:**
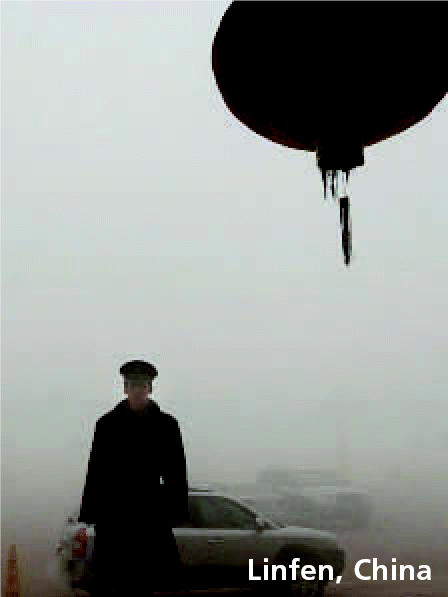


## Lead Lightens Up

Since 1993 the U.S. Department of Housing and Urban Development has awarded grants totaling approximately $1 billion for lead-based paint remediation in private low-income homes across the country. After six years, researchers led by Jonathan Wilson of the National Center for Healthy Housing looked at four such sites to measure the effectiveness of these programs. Their findings, published in the October 2006 issue of *Environmental Research*, show that dust lead levels on floors and window sills declined continuously over the six years since the intervention; levels of lead dust in window troughs, while gradually increasing over time, were still 75% lower than before the intervention.

## Hospitals Trigger Asthma

At least 20 million Americans suffer from asthma. Now the group Health Care Without Harm has released a report showing that hospitals abound with substances that can trigger or even cause asthma. These include cleaners such as disinfectants and sterilizers, fumes outgassing from building materials, pesticides, and latex gloves. Nurses are at particular risk from the disinfectant gluteraldehyde and the sterilizing agent ethylene oxide. Safer alternatives to these substances are described in the report, available at http://www.noharm.org/.

**Figure:**
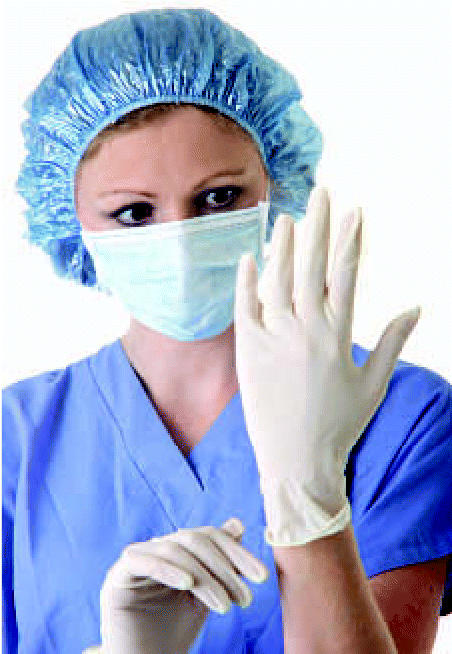


## Ballast Water Ballyhoo

Alien species of fish, mollusks, and aquatic weeds in U.S. waters—many introduced from ballast water discharged from ships—are believed to cost the nation nearly $8 billion. In a move to quell the further introduction of such species, a federal district judge in California ruled in September 2006 that the EPA must begin regulating ballast water as a biological pollutant beginning in 2008. Until now, the EPA has claimed a clause in the Clean Water Act exempts it from regulating discharges that are “incidental to the normal operations of a vessel.” The judge ruled, however, that this exemption is “plainly contrary to the congressional intent” of the act.

**Figure:**
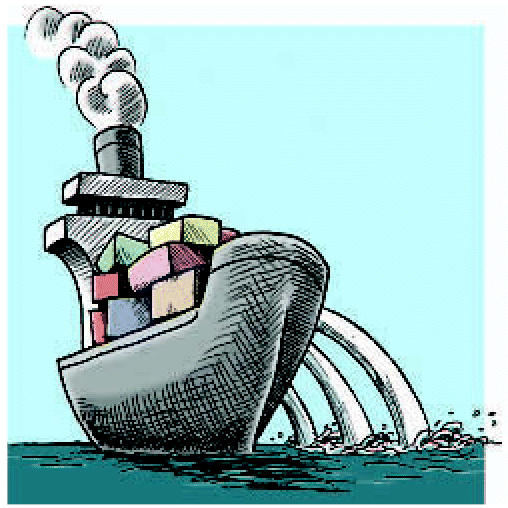


## The Ouch of ARGs

According to the WHO, more than 2 million Americans are infected each year with antibiotic-resistant pathogens, and 14,000 die as a result. A study in the 1 December 2006 issue of *Environmental Science & Technology* puts forth the idea that antibiotic resistance genes (ARGs)—pieces of DNA that make bacteria resistant to common antibiotics—should be considered emerging environmental contaminants. ARGs are rapidly spread by horizontal gene transfer among bacteria. Even if cells containing ARGs are killed, DNA released from them can spread to other cells. The study authors found tetracycline and sulfonamide ARGs in several types of waters in northern Colorado, including treated drinking water and recycled wastewater, both of which could be potential pathways for human exposure.

## Plateau in Smoking Rate Decline

A years-long decline in the adult smoking rate stalled between 2004 and 2005, according to the most recent report by the CDC. Reasons for the plateau may include smaller annual increases in cigarette prices, a 26.5% reduction in funding for comprehensive state programs in tobacco control and prevention between 2002 and 2006, and a doubling of tobacco company advertising and promotional spending between 1998 and 2003. The report also notes that 42.5% of current smokers stopped smoking for at least a day during the past year, and that just over half of all smokers successfully quit.

**Figure:**